# The impact of the COVID-19 pandemic on the well-being, work conditions, and education of early career psychiatrists in the WHO Eastern Mediterranean Region: study protocol

**DOI:** 10.3389/fpsyt.2024.1340181

**Published:** 2024-02-22

**Authors:** Seyedeh Reihaneh Hosseini, Ahmad Hajebi, Mohammadreza Shalbafan, Farnaz Ghannadi, Amine Larnaout, Marwa Nofal, Tomasz M. Gondek, Mariana Pinto da Costa

**Affiliations:** ^1^ Brain and Cognition Clinic, Institute for Cognitive Sciences Studies, Tehran, Iran; ^2^ Research Center for Addiction & Risky Behaviors (ReCARB), Psychosocial Health Research Institute, Iran University of Medical Sciences, Tehran, Iran; ^3^ Department of Psychiatry, School of Medicine, Iran University of Medical Sciences, Tehran, Iran; ^4^ Mental Health Research Center, Psychosocial Health Research Institute (PHRI), Department of Psychiatry, School of Medicine, Iran University of Medical Sciences, Tehran, Iran; ^5^ School of Medicine, Tehran University of Medical Sciences, Tehran, Iran; ^6^ Department of Psychiatry, Razi Hospital, Faculty of Medicine, University of Tunis El Manar, Tunis, Tunisia; ^7^ Helwan Mental Health Hospital, Cairo, Egypt; ^8^ Mental Health Research Network of Egypt, Cairo, Egypt; ^9^ Kingsley Green hospital, Hertfordshire partnership University NHS Foundation Trust, London, United Kingdom; ^10^ Iter Psychology Practices, Wroclaw, Poland; ^11^ Institute of Clinical Improvement, Warsaw, Poland; ^12^ Institute of Psychiatry, Psychology & Neuroscience, King’s College London, London, United Kingdom; ^13^ Institute of Biomedical Sciences Abel Salazar, University of Porto, Porto, Portugal

**Keywords:** COVID-19, early career psychiatrists, psychiatric trainees, work conditions, education, well-being, mental health, EMRO

## Abstract

**Background:**

The COVID-19 pandemic placed great strain on healthcare professionals, leading to a substantial impact and a redistribution of the workforce. Despite the active involvement of the Eastern Mediterranean Region Office (EMRO) and the World Health Organization in managing mental health crises, there is a knowledge gap concerning the working conditions and training opportunities available for early career psychiatrists (ECPs) during the pandemic period.

**Objectives:**

This study aims to investigate the impact of the COVID-19 pandemic on ECPs and how it affected their well-being, employment, and educational opportunities.

**Methods:**

A mixed methods study has been conducted in Iran, Egypt, and Tunisia, three EMRO member countries. It includes a cross-sectional survey with self-reported questions, and a qualitative study with individual in-depth interviews.

**Discussion:**

The findings of this study will raise awareness to the working conditions of ECPs within the EMRO region and its member societies, both during the COVID-19 pandemic and beyond. The results will serve as a basis for encouraging supervisors and policymakers to mitigate the pandemic’s impact on psychiatric training, strengthen healthcare systems’ preparedness, and equip early career psychiatrists with the necessary skills to deal with the mental health consequences of the COVID-19 pandemic.

## Introduction

Throughout the COVID-19 pandemic, healthcare employees faced exceptional work demands, resulting in a unique array of physical and psychological stressors. Reports indicate that these stressors elicited complex emotions, including dread, rage, anxiety, sorrow, and sleep difficulties (1, 2). In response to the COVID-19 health crisis, the World Health Organization (WHO) has prioritised the provision of mental health services at all population levels, addressing the psychological, emotional, and social needs of individuals and communities (3).

The Eastern Mediterranean Region Office (EMRO) has extensive expertise in providing psychosocial and mental health crisis support (4). The COVID-19 has stimulated further innovation, pushing for the development of new and effective mental health services (5). Previous research has showed that psychiatrists in various EMRO member countries have used different modalities of telepsychiatry as an alternative way of working, including online conferencing tools, individual and group psychotherapy, telephone hotlines or consultations (6). The abrupt transition to remote mental health service delivery has presented challenges in acquiring new technological skills, adapting to various modes of patient interaction, and navigating the complexities of providing adequate care through virtual platforms (7).

Early career psychiatrists (ECPs) have been notably affected by the pandemic, lacking practical learning opportunities, such as in-person clinical experiences and hands-on training, disrupting their traditional learning pathways. The lack of practical experience can impede the development of essential clinical competencies and skills (8). Reduced healthcare expenditures, decreased patient visits, and disruptions within healthcare systems have contributed to employment and financial insecurity. These financial stresses can impact psychiatrists’ mental health and career prospects, potentially leading to exhaustion and decreased job satisfaction (8).

The shortage of mental health professionals has worsened as several were redeployed to COVID-19 treatment facilities (9). Additionally, the insufficient availability of replacement personnel while individuals were isolated due to COVID-19 diagnosis resulted in an increased workload for the already operational workforce. This, in turn, significantly contributed to heightened levels of burnout among the existing staff. In a study examining the global repercussions of COVID-19 with 2707 participants from 60 different countries, it was revealed that 51% of healthcare workers reported feelings of burnout (10).

However, there is limited understanding of the working conditions of psychiatrists during and after the COVID-19 pandemic in EMRO countries. The unique challenges faced by healthcare systems in these disadvantaged communities, such as limited resources, insufficient mental health assistance, and restricted internet access, may have exacerbated the impact of the pandemic on mental healthcare professionals.

This study aimed to investigate how early career psychiatrists (ECPs) were exposed and impacted to the COVID-19 pandemic, including its effects on their well-being, work circumstances, and education.

## Methods

### Study design

A mixed-methods study will be conducted to investigate the impact of the COVID-19 pandemic on ECPs in three countries of the EMRO, a sub-community of WHO that comprises a group of developing countries with diverse economies located in Southwest Asia, Western Asia, and North Africa (11). This study will focus on three EMRO member countries: Tunisia, a North African nation with a lower-middle-income (12); Egypt, a country with a medium level of development with its Human Development Index (HDI) ranking of 101 out of 169 nations by the World Bank (13); and Iran, where the promotion of mental health and occupational mental health is often overlooked in psychiatry training programs (14).

To meet the eligibility criteria for participation in our study, individuals must: 1) be psychiatrists, having completed their specialization within the past five years, or psychiatric trainees (certified physicians undergoing psychiatric training at legitimate institutions in each country), 2) be employed in a clinical or academic setting through the pandemic, and 3) be willing to provide informed consent for their involvement in the study. The term early career psychiatrists (ECPs) refers to psychiatric trainees and psychiatrists who completed their training in the past five years.

### Phase 1: cross-sectional survey

#### Study instrument

A self-reported, 24-item, anonymous questionnaire has been developed in English for a worldwide cross-sectional study on the impact of the COVID-19 pandemic on the well-being, work conditions and education of early career psychiatrists (15). This will be translated into the respective languages of the participating nations via forward and backward translation.

The questionnaire covers: i) participants’ sociodemographic characteristics, ii) perspectives on COVID-19, iii) the impact of the pandemic on their professional careers, iv) impact on education, v) workplace environment, vi) well-being, and vi) the utilization of telepsychiatry in their respective countries.

#### Data collection

An online questionnaire with the corresponding URL will be distributed to potential participants in each country via email and social media. A non-random convenience sampling approach will be used.

#### Data analysis

Using IBM SPSS software, the collected survey data will be compiled into a single dataset and analyzed. Descriptive statistical analysis will summarize categorical variables using frequency distribution and continuous variables using means or medians. Cross-tabulations and chi-square tests will be used to investigate the links between sociodemographic factors and key outcomes. Multivariate analyses will identify predictors of professional well-being and job adaptation while considering confounding variables.

### Phase 2: qualitative study

#### Study instrument

Semi-structured individual interviews will be conducted, guided by a topic guide. Each interview is anticipated to last approximately one hour (in person or remotely). The topic guide details are provided in Appendix 1.

The interviews will be conducted by expert researchers with backgrounds in psychiatry or psychology, trained in qualitative research methods, and supervised by a senior qualitative researcher to ensure neutrality and minimise bias. The interview materials will be piloted to clarify content and maximize clarity.

#### Data collection

Purposive sampling (16) will be used to include participants from each country, continuing until reaching saturation, at which point no further novel information or insights arise in the interviews. All interviews will be audio-recorded.

This study will adhere to the best practice guidelines outlined in the Consolidated Checklist for Reporting Qualitative Research (COREQ) to maintain high-quality research standards (17).

#### Data analysis

The interviews will be transcribed verbatim. Initial codes, representing foundational pieces of information pertaining to the research question, will be generated. Patterns and relationships will be identified among the codes, leading to the classification of codes into categories known as themes. By understanding the core of each theme and how it represents different aspects of the data, we will give names and definitions to the themes. After exploring for possible sub-themes, we will thoroughly analyze the data through the Braun and Clarke thematic analysis (18). The research team will use the NVivo software for analysis.

The research team will review the themes and subthemes multiple times during the analysis process to ensure that there is internal consistency between the ideas conveyed and the codes used to generate each theme. The findings will be shared with participants for additional evaluation and validation.

## Ethics and dissemination

The assessment is anonymous, and participants will provide informed consent. We will obtain approval of the study from related review boards. Research Ethics Committees of Iran University of Medical Sciences approved the protocol of this study (IR.IUMS.REC.1401.769) to be in accordance with the ethical principles and national norms and standards for conducting Medical Research in Iran.

The findings will be published in scientific peer-reviewed journals as well as presented in international and local academic meetings.

## Discussion

Mental health conditions, such as depression, anxiety, sleep deprivation, and post-traumatic stress disorder, have been observed among physicians, nurses, and other healthcare workers due to the heightened stress levels associated with the COVID-19 pandemic (19). A cross-sectional survey in Europe reported how ECPs experienced significant changes in their working conditions and limited training opportunities during the COVID-19 pandemic (15). The European Psychiatric Association (EPA) conducted a cross-sectional survey of mental health professionals practicing in Europe and reported that the transition to remote psychiatric care resulted in fewer in-person consultations, with repercussions for patients lacking digital resources (20).

A cross-sectional study conducted in China revealed that medical personnel experienced anxiety and melancholy during the pandemic, and reported the potential benefits of psychological support and group therapy (21). In Iran, a cross-sectional study using an online questionnaire investigated the deleterious effects of COVID-19 on the education and mental health of general surgery and obstetrics/gynecology trainees (22). Similarly, in Italy, a survey (23) highlighted the negative impact of the pandemic on obstetrics and gynecology training programs and proposed innovative solutions to mitigate the deficiencies in training caused by COVID-19. An international survey covering 140 countries, with 6492 undergraduate and postgraduate medical learners, reported concerns regarding the quality of online education and the potential loss of educational experience during the pandemic (24). A narrative review investigated the mental health challenges encountered by healthcare workers and described factors such as long working hours, lack of social support, and insufficient protective measures (25).

Our study is targeting a diverse sample of participants and will include memory aids such as major event markers to enhance participant recall. Through the in-depth interviews we will further understand the challenges encountered by medical practitioners throughout the pandemic, and the role of the economical and geopolitical circumstances of each of the EMRO countries during this period.

By examining the impact of COVID-19 on psychiatric trainees and ECPs, this study aims to address the knowledge gap and offer insights into the experiences of this particular population in the EMRO region. Understanding the obstacles faced, the impact on their mental health, the adaptations to work conditions, and the disruptions to their educational and professional development can inform the development of targeted interventions and policies to meet their needs.

In particular, examining the work conditions and educational experiences of ECPs during the pandemic can shed light on potential systemic challenges and improvement opportunities within the mental healthcare systems of EMRO nations. These results can help healthcare administrators, mental health professionals and policymakers, to develop strategies to strengthen mental health services, address resource limitations, and amplify support for this important group of professionals.

## Ethics statement

This study was approved by the Ethics Committee of the Iran University of Medical Sciences. Participants provided their written informed consent to participate in this study.

## Author contributions

SH: Conceptualization, Investigation, Methodology, Writing – original draft. AH: Conceptualization, Methodology, Supervision, Writing – review & editing. MS: Conceptualization, Investigation, Methodology, Project administration, Supervision, Writing – review & editing. FG: Investigation, Writing – review & editing. AL: Investigation, Writing – review & editing. MN: Investigation, Writing – review & editing. TG: Conceptualization, Writing – review & editing. MP: Conceptualization, Methodology, Project administration, Supervision, Writing – review & editing.

## References

[1] Kang L Li Y Hu S Chen M Yang C Yang BX . The mental health of medical workers in Wuhan, China dealing with the 2019 novel coronavirus. Lancet Psychiatry Mar (2020) 7(3):e14. doi: 10.1016/s2215-0366(20)30047-x PMC7129673 32035030

[2] Zandifar A Badrfam R Khonsari NM Assareh M Karim H Azimzadeh M . COVID-19 and medical staff’s mental health in educational hospitals in Alborz Province, Iran. Psychiatry Clin Neurosci Sep (2020) 74(9):499–501. doi: 10.1111/pcn.13098 PMC7362126 32592626

[3] Organisation WH . Guidelines to help countries maintain essential health services during the COVID-19 pandemic. Available at: https://www.who.int/emergencies/diseases/novel-coronavirus-2019/technical-guidance/maintaining-essential-health-services-and-systems (Accessed 30 March 2020).

[4] Karam E El Chammay R Richa S Naja W Fayyad J Ammar W . Lebanon: mental health system reform and the Syrian crisis. BJPsych Int Nov (2016) 13(4):87–9. doi: 10.1192/s2056474000001409 PMC5619489 29093915

[5] Shah K Kamrai D Mekala H Mann B Desai K Patel RS . Focus on mental health during the coronavirus (COVID-19) pandemic: applying learnings from the past outbreaks. Cureus (2020) 12(3):e7405–5. doi: 10.7759/cureus.7405 PMC7182052 32337131

[6] Pereira-Sanchez V Adiukwu F El Hayek S Bytyçi DG Gonzalez-Diaz JM Kundadak GK . COVID-19 effect on mental health: patients and workforce. Lancet Psychiatry Jun (2020) 7(6):e29–30. doi: 10.1016/s2215-0366(20)30153-x PMC7239628 32445691

[7] Giordano L Cipollaro L Migliorini F Maffulli N . Impact of Covid-19 on undergraduate and residency training. surgeon: J R Colleges Surgeons Edinburgh Ireland (2021) 19(5):e199–206. doi: 10.1016/j.surge.2020.09.014 PMC7659986 33248923

[8] Eissazade N Hemmati D Ahlzadeh N Shalbafan M Askari-Diarjani A Mohammadsadeghi H . Attitude towards migration of psychiatric trainees and early career psychiatrists in Iran. BMC Med Educ Sep 22 (2021) 21(1):502. doi: 10.1186/s12909-021-02926-y PMC8459496 34551745

[9] Adiukwu F de Filippis R Orsolini L Gashi Bytyçi D Shoib S Ransing R . Scaling up global mental health services during the COVID-19 pandemic and beyond. Psychiatr Serv (Washington DC) (2022) 73(2):231–4. doi: 10.1176/appi.ps.202000774 34235945

[10] Morgantini LA Naha U Wang H Francavilla S Acar Ö Flores JM . Factors contributing to healthcare professional burnout during the COVID-19 pandemic: A rapid turnaround global survey. PloS One (2020) 15(9):e0238217. doi: 10.1371/journal.pone.0238217 32881887 PMC7470306

[11] Mirahmadizadeh A Fathalipour M Mokhtari AM Zeighami S Hassanipour S Heiran A . The prevalence of undiagnosed type 2 diabetes and prediabetes in Eastern Mediterranean region (EMRO): A systematic review and meta-analysis. Diabetes Res Clin Pract (2020) 160:107931. doi: 10.1016/j.diabres.2019.107931 31794806

[12] Spagnolo J Champagne F Leduc N Rivard M Piat M Laporta M . Mental health knowledge, attitudes, and self-efficacy among primary care physicians working in the Greater Tunis area of Tunisia. Int J Ment Health Systems. 2018/10/26 (2018) 12(1):63. doi: 10.1186/s13033-018-0243-x PMC6203218 30386422

[13] Elnemais Fawzy M . Mental health care in Egypt: Review of current state, policy, and needs. Int J Ment Health 2017/10/02 (2017) 46(4):339–45. doi: 10.1080/00207411.2017.1367447

[14] Eissazade N Shalbafan M Saeed F Hemmati D Askari S Sayed Mirramazani M . The impact of the COVID-19 pandemic on Iranian psychiatric trainees’ and early career psychiatrists’ Well-being, work conditions, and education. Acad. Psychiatry (2022) 46(6):710–7. doi: 10.1007/s40596-022-01674-5 PMC9217116 35732923

[15] Gondek T . Early career psychiatrists in europe during COVID-19 outbreak: Results of the EPA ECPC-EFPT cross-sectional survey. Eur Psychiatry (2021) 64(S1):S42–2. doi: 10.1192/j.eurpsy.2021.139

[16] Tongco MDC . Purposive sampling as a tool for informant selection. Ethnobot Res Appl (2007) 5:147–58.

[17] Tong A Sainsbury P Craig J . Consolidated criteria for reporting qualitative research (COREQ): a 32-item checklist for interviews and focus groups. Int J Qual Health Care (2007) 19(6):349–57. doi: 10.1093/intqhc/mzm042 17872937

[18] Braun V Clarke V . Using thematic analysis in psychology. Qual Res Psychol (2006) 3(2):77–101. doi: 10.1191/1478088706qp063oa

[19] de Filippis R El Hayek S Shalbafan M . Editorial: Mental illness, culture, and society: Dealing with the COVID-19 pandemic. Front Psychiatry (2022) 13:1073768. doi: 10.3389/fpsyt.2022.1073768 36405906 PMC9674302

[20] Rojnic Kuzman M Vahip S Fiorillo A Beezhold J Pinto da Costa M Skugarevsky O . Mental health services during the first wave of the COVID-19 pandemic in Europe: Results from the EPA Ambassadors Survey and implications for clinical practice. Eur Psychiatry: J Assoc Eur Psychiatrists (2021) 64(1):e41. doi: 10.1192/j.eurpsy.2021.2215 PMC8314055 34103102

[21] Dong Z-Q Ma J Hao Y-N Shen X-L Liu F Gao Y . The social psychological impact of the COVID-19 pandemic on medical staff in China: A cross-sectional study. Eur Psychiatry (2020) 63(1):e65. doi: 10.1192/j.eurpsy.2020.59 32476633 PMC7343668

[22] Moini A Maajani K Omranipour R Zafarghandi M-R Aleyasin A Oskoie R . Residency training amid the COVID-19 pandemic: exploring the impact on mental health and training, a lesson from Iran. BMC Med Educ (2021) 21(1):603. doi: 10.1186/s12909-021-03029-4 34872551 PMC8647502

[23] Bitonti G Palumbo AR Gallo C Rania E Saccone G De Vivo V . Being an obstetrics and gynaecology resident during the COVID-19: Impact of the pandemic on the residency training program. Eur J Obstet Gynecol Reprod Biol (2020) 253:48–51. doi: 10.1016/j.ejogrb.2020.07.057 32771888 PMC7395645

[24] Brown A Kassam A Paget M Blades K Mercia M Kachra R . Exploring the global impact of the COVID-19 pandemic on medical education: an international cross-sectional study of medical learners. Can Med Educ J Jun (2021) 12(3):28–43. doi: 10.36834/cmej.71149 PMC8263042 34249189

[25] Giorgi G Lecca LI Alessio F Finstad GL Bondanini G Lulli LG . COVID-19-related mental health effects in the workplace: A narrative review. Int J Environ Res Public Health (2020) 17(21):7857. doi: 10.3390/ijerph1721785 33120930 PMC7663773

